# M2 polarization of macrophages facilitates arsenic-induced cell transformation of lung epithelial cells

**DOI:** 10.18632/oncotarget.15232

**Published:** 2017-02-09

**Authors:** Jiajun Cui, Wenhua Xu, Jian Chen, Hui Li, Lu Dai, Jacqueline A. Frank, Shaojun Peng, Siying Wang, Gang Chen

**Affiliations:** ^1^ Department of Biochemistry, Medical College of Yichun University, Yichun, Jiangxi 336000, China; ^2^ Department Pharmacology & Nutritional Sciences, University of Kentucky College of Medicine, Lexington, KY 40536, USA; ^3^ Department of Neurology, Affiliated Provincial Hospital of Anhui Medical University, Hefei, Anhui 230001, China; ^4^ Department of Ultrasound, The First Affiliated Hospital of Xiamen University, Xiamen, Fujian 361001, China; ^5^ Department of Toxicology & Cancer Biology, University of Kentucky College of Medicine, Lexington, KY 40536, USA; ^6^ Department of Pathophysiology, School of Basic Medicine, Anhui Medical University, Hefei, Anhui 230032, China

**Keywords:** macrophage, lung cancer, arsenic, transformation

## Abstract

The alterations in microenvironment upon chronic arsenic exposure may contribute to arsenic-induced lung carcinogenesis. Immune cells, such as macrophages, play an important role in mediating the microenvironment in the lungs. Macrophages carry out their functions after activation. There are two activation status for macrophages: classical (M1) or alternative (M2); the latter is associated with tumorigenesis. Our previous work showed that long-term arsenic exposure induces transformation of lung epithelial cells. However, the crosstalk between epithelial cells and macrophages upon arsenic exposure has not been investigated. In this study, using a co-culture system in which human lung epithelial cells are cultured with macrophages, we determined that long-term arsenic exposure polarizes macrophages towards M2 status through ROS generation. Co-culture with epithelial cells further enhanced the polarization of macrophages as well as transformation of epithelial cells, while blocking macrophage M2 polarization decreased the transformation. In addition, macrophage M2 polarization decreased autophagy activity, which may account for increased cell transformation of epithelial cells with co-culture of macrophages.

## INTRODUCTION

Arsenic is a naturally existing element present in food, soil and water, and humans are exposed to arsenic through environmental contamination and occupational exposure. In contrast to the short-term, high dose therapeutic use of arsenic, chronic exposure to environmental levels of arsenic promotes skin, bladder, liver and lung cancers. The US Environmental Protection Agency reduced the permissible level of arsenic in drinking water from 50 ppb to 10 ppb in 2001. Still, arsenic is found over the permissible level in drinking water from private wells in many areas in western and southwestern states as well as in Alaska. Inorganic arsenic compounds promote human lung cancer and the primary route of arsenic exposure in humans is through diet and drinking water.

Our previous work showed that long-term arsenic exposure induces transformation of lung epithelial cells *in vitro*, and xenograft tumor formation in nude mice. Our data indicated that oxidative stress was a driving force to promote, and autophagy was a cell self-protective mechanism against, the carcinogenic actions of arsenic [1]. We later determined that arsenic increased the secretion of inflammatory cytokines by the epithelial cells, which in turn may facilitate arsenic-induced transformation of epithelial cells by inhibiting autophagy [2].

Macrophages, especially alveolar macrophages (AM), are the major immune cell population in the lung, and regulate the local microenvironment by orchestrating immune responses under both physiological and pathological conditions [3]. AM secret a wide variety of soluble factors, such as cytokines, chemokines, growth factors and enzymes, and the effector functions of these factors extend far beyond phagocytosis and antigen presentation. In addition, cells of the monocyte-macrophage lineage are diverse with high plasticity. The phenotype and function of AM are altered in patients with lung cancer [4]. Therefore, this study investigated crosstalk between macrophages and lung epithelial cells and examined the effects of arsenic carcinogenicity in the co-culture system.

Macrophages carry out diverse effector functions after activation. Depending on the environmental cues, the activation of macrophages can be classified as classical (or M1) or alternative (or M2). The phenotype of M1 macrophages includes: secretion of pro-inflammatory cytokines, high production of reactive nitrogen with strong microbicidal and high tumoricidal activity. In contrast, M2 macrophages promote tissue remodeling, tumor progression and are immune suppressive. M1 and M2 macrophages are also distinct in their cellular membrane markers, cytokine and chemokine expression profiles as well as arginine metabolism. For instance, M1 macrophages express high levels of CD86, produce pro-inflammatory cytokines, such as TNF-α and IL12, and chemokines, such as CXCL11. In contrast, M2 macrophages express high levels of CD163, CD204 or CD206 on their membrane, produce high levels of cytokine IL-10, and chemokines, CCL17 and CCL18. In rodents, high levels of inducible nitric oxide synthases (iNOS) and arginase-1 (Arg-1) were observed in M1 and M2 macrophages, respectively [5–7]. Macrophage polarization may alter a microenvironment to either promote or inhibit lung tumor formation.

In our current study, we used a co-culture system of BEAS-2B cells, a human lung epithelial cell line, with macrophages derived from THP-1 cells. We investigated how arsenic regulates macrophage activation in the presence or absence of the epithelial cells, and how an alteration in macrophage activation status in turn affects arsenic-induced cell transformation. THP-1 is a human monocyte cell line, widely used as an *in vitro* model system to study macrophage functions [8]. Our data suggest the existence of a crosstalk between macrophages and epithelial cells. Long-term arsenic exposure polarizes macrophages towards M2 activation through ROS generation; co-culture of epithelial cells further enhances this macrophage polarization. More importantly, macrophage M2 polarization in turn facilitates arsenic-induced transformation of epithelial cells by inhibiting autophagy activity in these cells. Blocking macrophage M2 polarization decreases arsenic-induced transformation. The results provide new insights into how macrophages regulate the microenvironment in arsenic-induced lung carcinogenesis.

## RESULTS

### Co-culture of THP-1 derived macrophages enhances arsenic-induced transformation of BEAS-2B (B2B) cells

**Figure 1:**
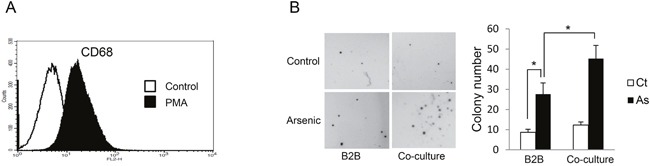
Co-culture with THP-1 derived macrophages enhances arsenic induced transformation of B2B cells **A**. CD68^+^ THP-1 cells were significantly increased 24 hours after 50 nM PMA treatment as shown by flow cytometric analysis. **B**. B2B cells alone or co-cultured with THP-1 derived macrophages were exposed to 0.25 μM arsenic for 12 weeks and arsenic-induced cell transformation of B2B cells was determined by soft agar assay. The experiment was performed in triplicate. * indicates *P < 0.05*.

Our previous work showed that exposure of B2B cells, which are immortalized human lung branchial epithelial cells, to 0.25 μM sodium arsenite for 12 weeks induced transformation as evidenced by anchorage-independent cell growth (colony formation) [1]. To determine the effect of macrophages on arsenic-induced transformation of lung epithelial cells in this current study, we co-cultured B2B cells with macrophages using transwell plates; THP-1-derived macrophages were placed in the upper compartments and B2B cells in lower compartments. Macrophages were derived from THP-1 cells (a human monocyte cell line) after treatment with 50 ng/mL of PMA for 24 hours; this system is an *in vitro* model widely used for macrophage study [8]. The newly generated macrophages are in a resting stage and a categorized as M0 status [9]. As shown in Figure 1A, the differentiation of THP-1 toward the macrophage phenotype was confirmed by the induction of CD68, a marker for macrophages differentiation [8]. After exposure to arsenic for 12 weeks, cell transformation of epithelial cells was determined by soft agar assay. The results indicate that co-culture of macrophages significantly enhanced arsenic-induced cell transformation of B2B cells as colony numbers increased from 27.67 ± 5.51/well in control to 45.33 ± 6.51/well with co-culture, *P* < *0.05* (Figure 1B).

### Arsenic induces alternative activation of THP-1-derived macrophages, which is further enhanced by co-culture with B2B cells

**Figure 2:**
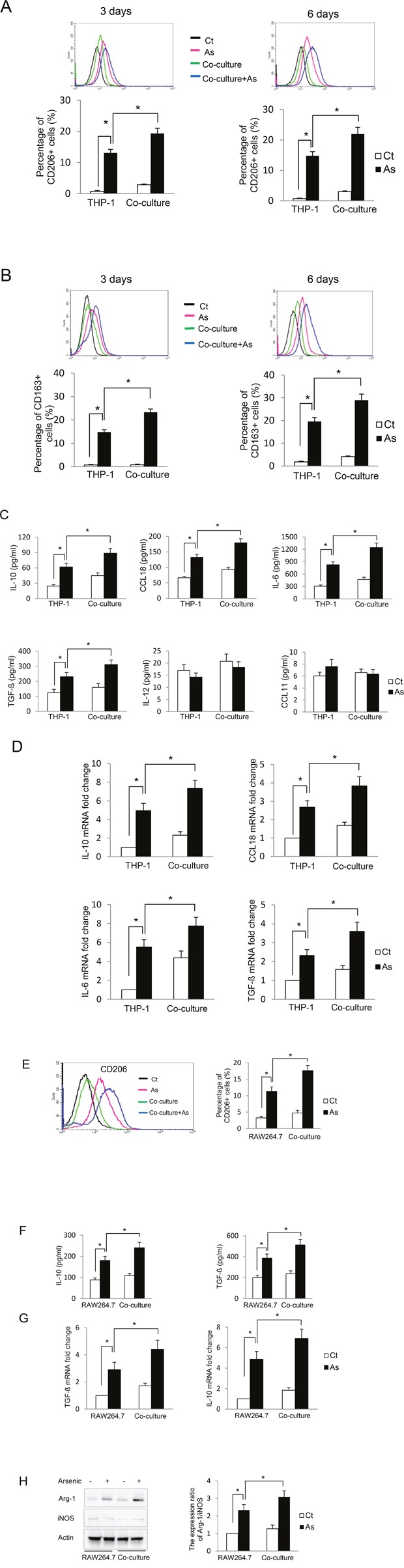
Arsenic induces THP-1 macrophage M2 polarization which is further enhanced by co-culture with lung epithelial cells THP-1 macrophages alone or co-cultured with B2B cells were treated with or without arsenic for 3 or 6 days. CD206^+^ and CD163^+^ macrophages were evaluated by flow cytometry **A-B**. After 6 days of treatment, the secretion levels of IL10, IL6, CCL18, TGF-β, IL12 and CXCL18 were determined by ELISA. **C**. and the mRNA levels of IL10, IL6, CCL18 and TGF-β in the macrophages were determine by RT-PCR **D**. RAW264.7 cells were cultured alone or co-cultured with E10 cells and exposed to 0.25 μM sodium arsenite for 6 days. CD206^+^ RAW264.7 cells were evaluated by flow cytometry. **E**. The secretion of IL10 and TGF-β in the media was evaluated by ELISA **F**. and the mRNA levels of IL10 and TGF-β in the RAW264.7 cells were determined by RT-PCR **G**. Arg-1 or iNOS protein levels in RAW264.7 cells were determined by western blot, quantified and the ratio of Arg-1 vs iNOS protein levels is shown **H**. All experiments were performed in triplicate. * and ** indicate *P < 0.05* and *P < 0.01* respectively.

To determine the effect of arsenic on macrophage activation, we first examined the expression of CD163 and CD206, two markers for alternative-activated macrophages [6]. As shown in Figure 2A and 2B, arsenic increased the expression of both CD163 and CD206 on the macrophages, and co-culture of B2B cells further increased the proportion of the macrophages expressing these markers (*P* < *0.05*). It has been shown that secretion levels of IL10, TGF-β and CCL18 are enhanced when macrophages are alternatively activated, while increased levels of IL12 and CXCL11 are observed in classically activated macrophages [5]. To confirm the phenotype of the arsenic-induced activated macrophages, the secretion levels of IL10, TGF-β, CCL18, IL12 and CXCL11 were determined. Arsenic exposure increased the secretion of IL10, TGF-β and CCL18 in the media (*P* < *0.01*); co-culture of B2B cells further increased these levels (*P* < *0.05*). The levels of IL12 and CCL11 did not change significantly (Figure 2C). The increased secretion of these cytokines by the macrophages was further supported by their increased mRNA levels in the macrophages (*P* < *0.05*) (Figure 2D). Another important marker for macrophage M2 activation is the protein level ratio of arginase-1 (Arg-1) verses inducible nitric oxide synthesis (iNOS), but this parameter has been noted to be more definitive in mouse macrophages [7]. To further confirm the effect of arsenic on macrophage polarization and explore whether this effect is specific to THP-1-derived macrophages, Raw 264.7 cells (a mouse macrophage cell line) and E10 cells (immortalized mouse lung epithelial cells) were cultured together and exposed to arsenic. Arsenic exposure not only up-regulated CD206 and increased secretion of IL10 and TGF-β, but also increased the ratio of Arg-1 to iNOS in Raw 264.7 cells (*P* < *0.05*) (Figure 2E-2H), indicating that arsenic exposure skewed macrophages toward the M2 phenotype. Co-culture of E10 cells further enhanced arsenic-induced macrophage M2 polarization. More importantly, the effects of arsenic and co-culture of epithelial cells on macrophage polarization is not specific to THP-1 cells.

### Inhibition of macrophage alternative activation by lipopolysaccharides (LPS) plus interferon gamma (IFN-γ) decreases arsenic-induced B2B cell transformation

**Figure 3:**
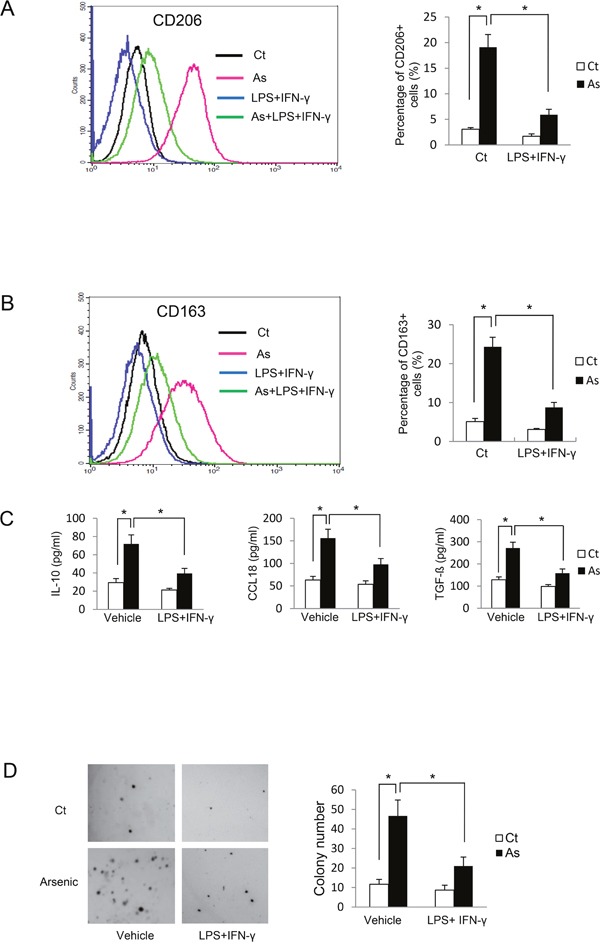
Inhibition of M2 polarization by LPS plus IFN-γ suppresses arsenic B2B transformation THP-1 cells were co-cultured with B2B cells and exposed to arsenic with or without co-treatment of LPS plus IFN-γ for 6 days. THP-1 macrophages with CD206 **A**. and CD163 **B**. expression were determined by flow cytometry and the secretion levels of IL10, CCL18 and TGF-β were determined by ELISA **C**. The co-culture was maintained for 12 weeks and cell transformation of B2B cells was determined by soft agar assay. D. The experiment was performed in triplicate. * and ** indicate *P < 0.05 P < 0.01*, respectively.

LPS and IFN-γ together promote classical macrophage activation and inhibit alternative activation of THP-1-derived macrophages [9]. To confirm the important role of alternative activation of macrophages on arsenic-induced B2B cell transformation, arsenic-induced cell transformation was assessed after co-treatment of B2B cells with macrophages treated with or without LPS plus IFN-γ. As shown in Figure 3A-3C, co-treatment of LPS plus IFN-γ inhibited alternative activation of macrophages, as evidenced by decreased levels of CD206, CD163, IL10, CCL18 and TGF-β (*P* < *0.05*). More importantly, arsenic-induced cell transformation was also inhibited by LPS plus IFN-γ; colony numbers were decreased from 46.67 ± 8.14/well without LPS and IFN-γ to 21.00 ± 4.58/well with LPS and IFN-γ (*P* < *0.05*) (Figure 3D). These data confirm a promoting role of macrophage M2 activation on arsenic-induced cell transformation.

### Autophagy in B2B cells is inhibited by co-culture with M2 macrophages

**Figure 4:**
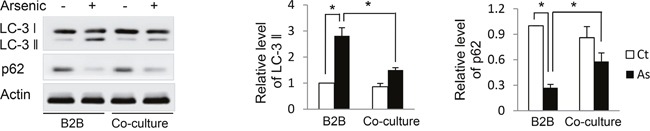
Co-culture with THP-1 M2 macrophages inhibits autophagy activity in B2B cells B2B cells were co-cultured with and without THP-1 macrophages in the presence or absence of arsenic for 12 weeks. Cells were harvested and LC-3 and p62 levels were determined by western blot. The relative protein levels were quantified using Imagine J software. The experiment was performed in triplicate. * and ** indicate *P < 0.05 P < 0.01*, respectively.

Our previous study established that oxidative stress is the driving force for, and autophagy acts as a cell self-protective mechanism against, arsenic-induced cell transformation [1]. The compromised autophagic activity that accompanies long-term arsenic exposure, which is evidenced by decreased LC3-II and increased P62 levels, contributes to arsenic-induced cell transformation. Therefore, in current study we investigated whether co-culture of macrophages with alternative activation affects autophagy activity in B2B cells. As shown in Figure 4, arsenic decreased LC3-II and increased P62 levels in B2B cells in the co-culture comparing to arsenic-treated B2B alone (*P* < *0.05*), indicating autophagy activity in B2B cells was inhibited by the co-culture of M2 macrophages.

### ROS promotes alternative activation of macrophages

**Figure 5:**
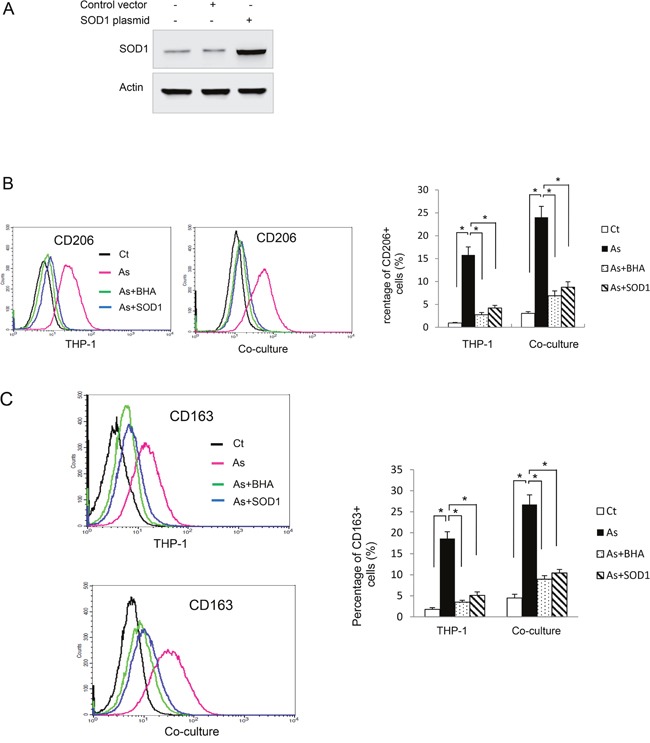
Reactive oxygen species (ROS) mediates arsenic induced THP-1 macrophage M2 polarization **A**. THP-1 macrophages were transfected with SOD1 plasmid and the level of SOD1 was determined by western blot. THP-1 or SOD1-overexpressing THP-1 macrophages were exposed to arsenic with or without co-treatment of BHA for 6 days and CD206 **B**. and CD163 **C**. positive macrophages were evaluated by flow cytometry. The experiment was performed in triplicate. * and ** indicate *P < 0.05 P < 0.0 1*, respectively.

We next sought to determine how arsenic skewed macrophages towards M2 phenotype. It has been shown that ROS induces macrophage alternative activation [5]. It is also well known that arsenic induces ROS generation in B2B and other cells [1, 10]. To determine whether ROS plays a role in arsenic-promoted alternative activation of macrophages, we transfected THP-1 derived macrophages with the SOD1 plasmid. SOD1 is an important anti-oxidant enzyme and over-expression of SOD1 decreases ROS generation in B2B cells [1]. Over-expression of SOD1 in these cells was confirmed by western blot (Figure 5A). The levels of CD106 and CD263 on the macrophages as well as secretion levels of IL-10 and CCL18 induced by arsenic were determined with or without co-treatment of BHA, a potent anti-oxidant [5]. As shown in Figure 5B-5C, over-expression of SOD1 or co-treatment of BHA significantly inhibited arsenic-induced alternative activation of macrophages, as evidenced by decreased expression of CD106 and CD263 (*P* < *0.01*).

## DISCUSSION

Although the cell mono-culture model has provided much of our current understanding of how single lineages of cells responds to stimuli, such as arsenic, it reflects few intercellular communications among the variety of cell types that exist in organs. Therefore, co-culture of multiple cell types is being used with increasing frequency as a solution to bridge this gap. In the current study, we used an *in vitro* co-culture model to investigate the crosstalk between epithelial cells and macrophages and to study the carcinogenic effects of arsenic.

Most studies that investigate arsenic carcinogenicity have focused on the carcinogenic effects of arsenic on tissue cells. For example, our previous work determined that long-term arsenic exposure induces transformation of lung epithelial cells [1, 2]. Although cell transformation *per se* is a critical step of tumor initiation, additional alterations in the microenvironment that surround the transformed cells are indispensable for the initiation and development of a lung tumor [11]. For this reason cancer has been suggested as a systemic disease [12] and, to better understand it, we must not only study the cancer cells, but the cancer cells together with the microenvironment in which the cancer cells initiate and grow.

A key component of the microenvironment is the immune system. [11], and in the lung, macrophages are the major immune cells. Macrophages, which are very heterogeneous and highly plastic, are subtly controlled by small changes in the microenvironmental signals. In tissues, the phenotype and functions of macrophages are constantly changed; they may undergo classical M1 activation or alternative M2 activation in response to environmental cues [13]. In addition, it was shown that the phenotype of polarized M1 or M2 macrophages can be reversed *in vitro* and *in vivo* [14, 15]. The M1/M2 states mirror the Th1/Th2 polarization of T helper cells. M1/Th1 and M2/Th2 phenotypes are dominant in pro- and anti-tumor microenvironment, respectively. Therefore, the crosstalk between macrophages and lung tissue cells, such as epithelial cells, may determine a microenvironment that is pro- or anti-tumorigenesis. Advanced study has shown that M2 polarization of macrophages promotes lung tumor formation [16].

The cytotoxic effects of arsenic on macrophages have been reported [17–19]. However, whether chronic arsenic exposure alters the functions of macrophage and, if it does, how this alteration is related to arsenic tumorigenicity in the lungs remains a mystery. The communication among cells can be complicated; a crosstalk between transformed/cancer cells and macrophages, through soluble mediators, such as cytokines and chemokines, has been shown to be critical for lung cancer development [3, 20]. In the present study, which focused on the crosstalk through soluble mediators, our data show that long-term arsenic exposure polarizes macrophages towards M2 phenotype; co-culture with epithelial cells further enhances this polarization. It should be noted that B2B cells constitutively secreted TGF-β[21], which could account for a minor M2 polarization in the co-culture in the absence of arsenic (Figure 2A, 2B). In addition, ROS plays a critical role in M2 polarization since inhibition of ROS generation ameliorated the polarization [22, 23]. Furthermore, long-term arsenic exposure changes the soluble mediators in the media, which inhibits autophagic activity in the epithelial cells and facilitates arsenic-induced transformation of these cells. IL-6 is likely an important mediator that inhibits autophagy and enhances cell transformation since IL-6 was increased in the co-culture system (Figure 2C) and our previous work has shown IL6 inhibits autophagy and enhances transformation of B2B cells [2]. Finally, the current study focuses on how an interaction between macrophages and epithelial cells contributes to arsenic-induced transformation of the epithelial cells. In the future study, it would be very interesting to determine the phenotypic, biochemical and metabolic changes in these cells after their transformation.

## MATERIALS AND METHODS

### Materials

Sodium arsenite solution was purchased from Merck. Phorbol 12-myristate 13-acetate (PMA) and butylated hydroxyanisole (BHA) were purchased from Sigma Aldrich. Anti-human CD68 antibody conjugated with phycoerythrin (PE), CD163 antibody conjugated with FITC and anti-mouse CD206 antibody conjugated with PE were from Biolegend. Anti-human CD206 antibody conjugated with FITC was from BD Biosciences. SOD1 DNA plasmids (NM_001752 and NM_000454) were purchased from Origene. Antibodies against LC-3II (Medical and Biological Laboratories), p62 (Sigma Aldrich), Arg-1 (Santa Cruz) and iNOS (Millipore) were used.

### Cell culture

BEAS-2B (B2B, immortalized human bronchial epithelial cells), THP-1 (a human monocytic leukemia cell line) and RAW264.7 (a mouse macrophage cell line) were purchased from American Type Culture Collection (ATCC). Cell lines were authenticated on the basis of viability, recovery, growth, and morphology. B2B cells were cultured in BEGM (Lonza) and THP-1 cells were cultured in 1640 medium containing 10% FBS. RAW264.7 cells were cultured in DMEM medium containing 10% FBS. E10 (a mouse lung alveolar epithelial cell line) was kindly provided by Dr. Maria Ramirez (Pulmonary Center, Boston University School of Medicine) and were cultured in CMRL medium containing 10% FBS (Hyclone) as previously reported [24]. All cells were maintained at 37°C with 5% CO2 in tissue culture incubators. Sodium arsenite solution was used for arsenic treatment.

### Differentiation of THP-1 monocytes into macrophages

The method for differentiation of THP-1 cells into macrophages has been reported previously. [8] Briefly, the macrophages were generated by treatment of THP1-cells with 50 ng/mL PMA for 24 h; cells were subsequently washed with PBS to remove PMA. THP-1 macrophage differentiation was verified by monitoring the macrophage differentiation marker CD68 (Biolegend, Cat 333805) by flow cytometry.

### Co-culture experiments and arsenic treatment

THP-1-derived macrophages were co-cultured with B2B cells in transwell plates (Costar, Cat 3412) with a 2:1 ratio as reported previously [21]. Briefly, 10 × 10^5^ THP-1 macrophages were seeded onto 0.4 μm pore inserts (upper compartments) and 5 × 10^5^ B2B cells were seeded in the bottom compartments of the transwell plates. For arsenic treatment, cells in both the upper and bottom compartments were exposed to 0.25 μM sodium arsenite [1]. B2B cells were passaged and re-plated into the bottom wells every week as described previously [1]. The inserts were replaced every week with newly generated THP-1 macrophages since apoptosis was observed with longer culture. Co-cultures were maintained for 12 weeks to induce B2B cell transformation as described previously [1]. For the co-culture of mouse macrophages and lung epithelial cells, 10 × 10^5^ RAW264.7 macrophages were seeded onto 0.4 μm pore inserts and 5 × 10^5^ E10 cells were seeded in the bottom compartments of the transwell plates. Cells were treated with 0.25 μM sodium arsenite and maintained for 6 days.

### Soft agar assay

B2B cell transformation was determined by anchorage-independent growth in soft agar as described previously [1]. Briefly, 5 × 10^3^ cells were plated on six-well plates containing a bottom layer of 0.6% low- temperature-melting agar in BEGM media, and a top layer of 0.3% agar in BEGM media. The top layer of agar was covered with 1 mL of culture medium with medium replacement every 3 days. Colonies per well were counted and photographed after 3 weeks of growth.

### Flow cytometry assay

The expression of CD68, CD163 and CD206 on the membrane of macrophages was determined by flow cytometry using the protocols provided by the manufacturer (BD Bioscience). Briefly, THP-1-derived macrophages were harvested then stained with CD68 antibody conjugated with PE, CD163 antibody conjugated with FITC and CD206 antibody conjugated with FITC, followed by flow cytometry analysis with a BD Biosciences Digital LSR II. Data were analyzed using FlowJo software (Tree Star Inc.).

### Plasmid and transfection

Transfection of SOD1 DNA plasmid has been reported previously [1]. Briefly, macrophages were transfected with plasmids using Lipofectamine 2000 (Invitrogen) and selected by G418 (Invitrogen) according to the manufacturer's protocol. The transfection was confirmed by immunoblot analysis.

### Western blot analysis

Western blots was performed as described previously [1]. Briefly, aliquots of the protein samples (20-40 μg) were separated by electrophoresis and transferred to nitrocellulose membranes. After blocking, the membranes were probed with primary antibodies and then with secondary antibody conjugated to horseradish peroxidase. The immune complexes were detected by the enhanced chemiluminescence method (PerkinElmer).

### RT-PCR assay

Quantitative real-time reverse transcription-PCR was performed as described previously [2]. Briefly, total RNA was extracted with TRIzol reagent (Invitrogen, 15596-026) and was reverse transcribed to cDNA using a Reverse Transcription System (Promega, A3500) according to the manufacturer's instructions. PCR was performed on a Lightcycler 480 system (Roche) using a Power SYBR Green PCR Master kit (Invitrogen, 4368706). The standard -ΔΔCt method was used for determining changes in gene expression. The relative expression level of a given mRNA was assessed by normalizing to the housekeeping gene, beta-actin, and comparing with control values. The primers used for analysis were as below: IL10 forward, 5′-gcctaacatgcttcgagatc-3′; IL10 reverse, 5′-tgatgtctgggtctttc-3′; IL6 forward, 5′-aacctgaaccttccaaagatgg-3′; IL6 reverse, 5′-tctggcttgttc ctcactact-3′; CCL-18 forward, 5′-tctatacctcctggcagattc-3′; CCL-18 reverse, 5′-tttctggacccacttcttat-3′; TGF-β forward,5′-ggtacctgaacccgtgttgct-3′; TGF-β reverse,5′-tgttgctgtatttctggtaacagctc-3′; Beta-actin forward, 5′-agcac agagcctcgccttt-3′; beta-actin reverse, 5′-agggtgaggatgc ctctctt-3′, Mouse TGF-β forward, 5′-tgacgtcactggagttgac gg-3′; mouse TGF-β reverse, 5′-ggttcatgtcatggatggtgc-3′, Mouse IL-10 forward,5′- tggcccagaaatcaaggagc-3′; Mouse IL-10 reverse, 5′-cagcagactcaatacacact-3′, Mouse beta-actin forward, 5′-tgtgatggtgggaatgggtcag-3′; Mouse beta-actin reverse, 5′-tttgatgtcacgcacgatttcc-3′.

### Measurement of secreted cytokines

Culture supernatants were collected and centrifuged at 1,000 rpm for 5 min. The cytokine levels were measured for IL-6, IL-10, IL-12, TGF-β, CCL11 and CCL18 with Platinum ELISA kits (eBioscience) according to the manufacturer's instructions. Cytokine measurements were determined using SpectraMax M2 reader (Molecular Device) and analyzed using SoftMax PRO 4.0 software.

### Measurement of autophagy activity

Autophagy activity upon arsenic exposure was monitored using a combination of LC3-II and P62 levels. Arsenic at 0.25 μM increased LC3-II, which has been verified as a result of enhanced autophagy activity in our previous work [1]. To confirm that the alteration of LC3-II reflected the change of autophagic flux, the expression levels of P62 (SQSTM1) were also evaluated. P62 is an autophagosome membrane associated protein and is degraded in lysosomes after autophagosomes fuse with lysosomes; thus its level is negatively regulated by autophagic flux [2].

### Statistical analysis

Differences among treatment groups were evaluated by ANOVA. Presented data are the mean ± SEM of three experiments. *P* < 0.05 was considered statistically significant. In cases in which significant differences were detected, specific *post-hoc* comparisons between treatment groups were examined by Student–Newman–Keul tests.
